# Life-Threatening Delayed Pericardial Tamponade Following Blunt Chest Trauma

**DOI:** 10.7759/cureus.81578

**Published:** 2025-04-01

**Authors:** Brian Musch, Rachel A Daley, Alyssa McMandon, Saptarshi Biswas

**Affiliations:** 1 Surgery, Grand Strand Medical Center, Myrtle Beach, USA; 2 Medicine, Edward Via College of Osteopathic Medicine, Spartanburg, USA

**Keywords:** blunt chest trauma, delayed pericardial tamponade, level one trauma, motor vehicle collision, polytrauma scenario

## Abstract

Pericardial tamponade, although rare, is a life-threatening complication of trauma. Typically occurring acutely after penetrating injuries, it can also present in a delayed fashion following blunt trauma, posing diagnostic and therapeutic challenges. We report a rare case of a 33-year-old male who developed delayed pericardial tamponade two weeks after sustaining severe multisystem injuries in a motor vehicle accident. This case emphasizes the need for close monitoring for an extended period of time as delayed complications do occur in severe polytrauma scenarios.

## Introduction

Pericardial tamponade, commonly associated with penetrating chest trauma, can also arise after blunt injuries. Tamponade physiology arises when fluid accumulates in the pericardial sac, impairing venous return to the heart, ventricular contractility, and cardiac output [1]. Key clinical findings in patients with tamponade are hypotension, elevated jugular venous pressure, and muffled heart sounds [2]. However, in acute situations in the trauma bay, they are often difficult to diagnose. Without prompt intervention, it may progress to cardiogenic shock and death [3].

Delayed-onset pericardial tamponade presents significant diagnostic and therapeutic challenges due to its nonspecific symptoms such as chest pain, shortness of breath, tachycardia, and hypotension. In trauma patients with ongoing or delayed complications, these signs may be misinterpreted as myocardial contusion, pulmonary embolism, or hemorrhagic shock, emphasizing the need for early imaging and multidisciplinary care to ensure timely diagnosis and prompt intervention [4].

We present a rare case of a 33-year-old male who sustained high-velocity blunt polytrauma from a motor vehicle accident. Despite initial stabilization, he developed delayed pericardial tamponade two weeks later, highlighting the critical need for vigilance in recognizing and managing delayed trauma-related complications.

## Case presentation

A 33-year-old male was airlifted to the trauma center as a Level 1 activation following a high-speed motor vehicle crash. He was intubated in the field by emergency medical services for airway protection. Upon arrival at the trauma bay, the patient was tachycardic, hypotensive, and hypoxic (O₂ saturation 83%), with a Glasgow Coma Scale score of 3. Primary and secondary surveys were initiated per the Advanced Trauma Life Support protocol.

**Figure 1:**
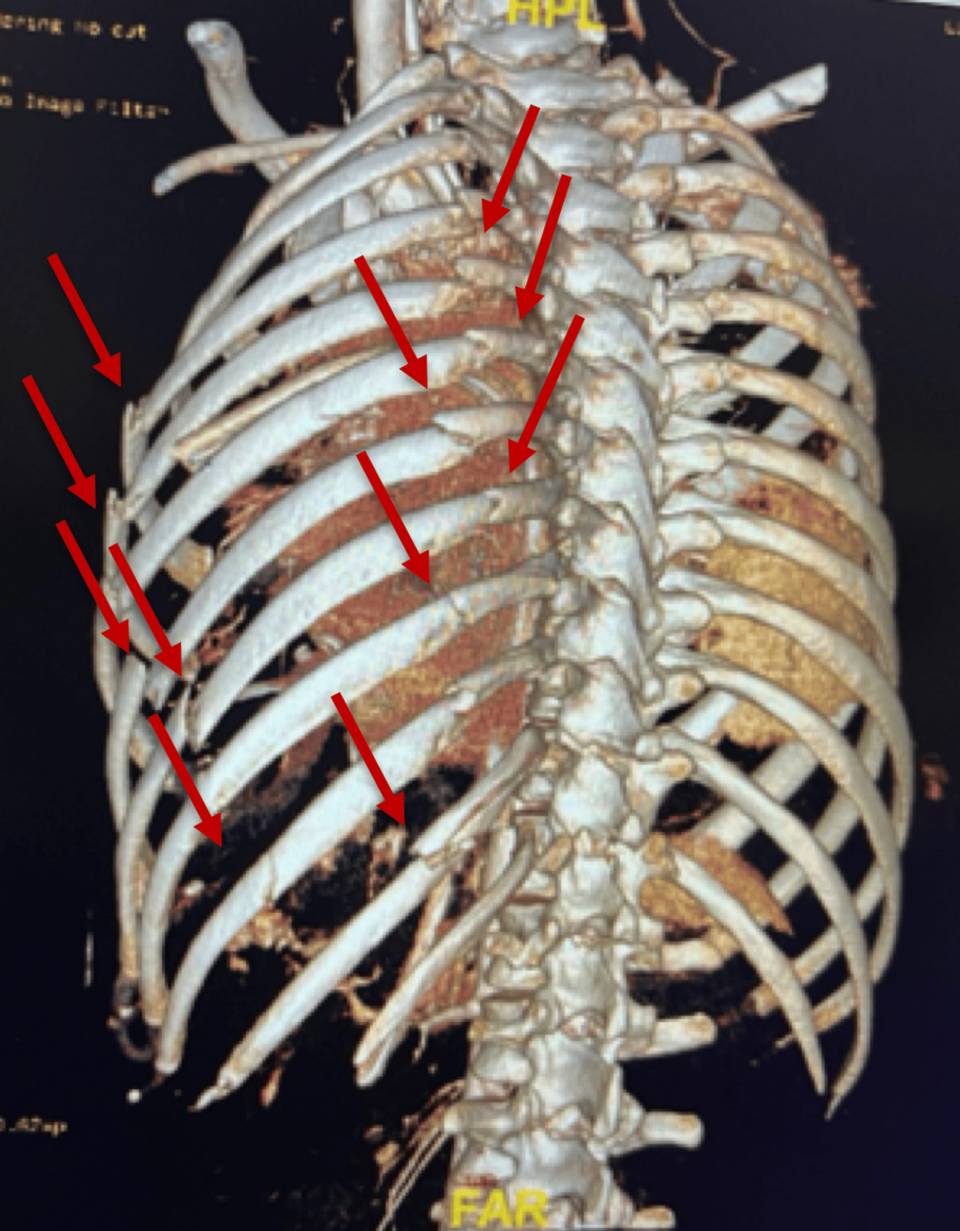
3D reconstruction revealing flail chest The red arrows indicate multiple rib fractures and a flail chest.

**Figure 2:**
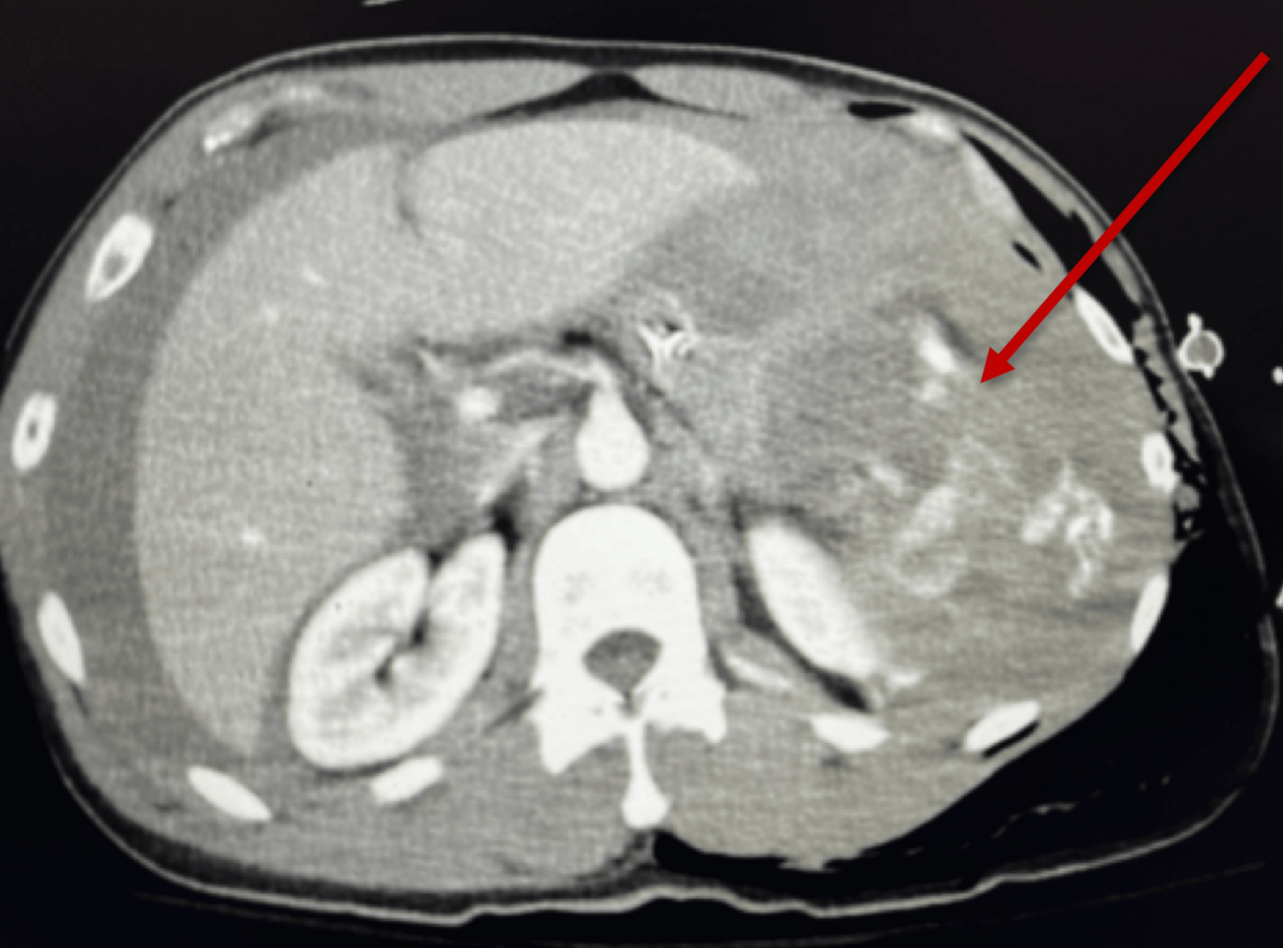
Grade IV splenic laceration The red arrow shows splenic rupture.

Extended focused assessment with sonography for trauma was positive for right upper quadrant free fluid and negative lung sliding. He received tranexamic acid and was started on hemodynamic resuscitation, followed by administration of blood products. Chest X-ray and pelvic X-ray revealed multiple rib fractures with pulmonary contusion, along with a pelvic fracture. A CT scan confirmed acute left-sided fractures of the second to 11th ribs, a moderate-sized pneumothorax, and a comminuted clavicle fracture. An anatomical flail chest was detected on the left third to 10th ribs, with significant displacement involving the fourth to ninth ribs (Figure 1). Pneumomediastinum and extensive subcutaneous emphysema were noted extending along the left chest and neck walls. The spleen was shattered (Figure 2), showing no enhancement, along with a large volume of hemoperitoneum. A comminuted fracture of the left superior pubic ramus extended into the anterior wall of the acetabulum, accompanied by a nondisplaced fracture of the left inferior pubic ramus. The patient was emergently taken to the operating room, where he underwent an exploratory laparotomy, splenectomy, and damage control surgery.

In the following week, the patient underwent multiple orthopedic surgeries, including open reduction and internal fixation of the ribs, along with video-assisted thoracoscopic surgery and cryoablation, all of which were uneventful. The postoperative course was marked by elevated troponins peaking at 23 ng/mL, consistent with a suspected myocardial contusion, which normalized over time. A blunt cardiac trauma workup was initiated, along with a cardiology consultation.

**Figure 3:**
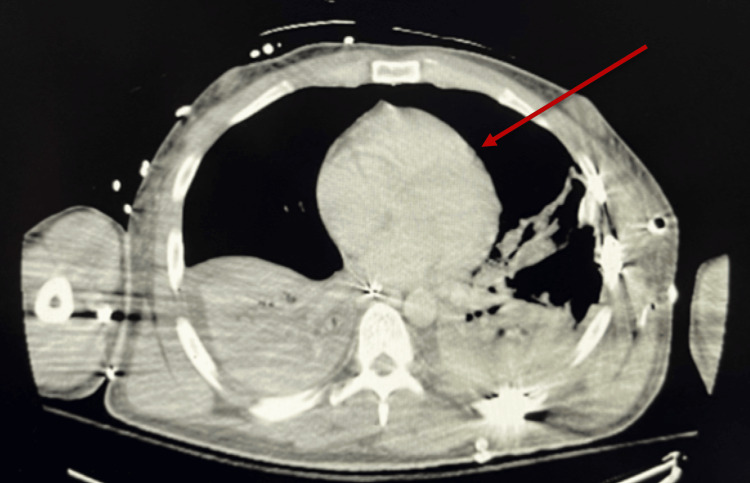
Chest CT on admission showing no evidence of pericardial effusion The red arrow shows normal pericardium and lack of effusion.

**Figure 4:**
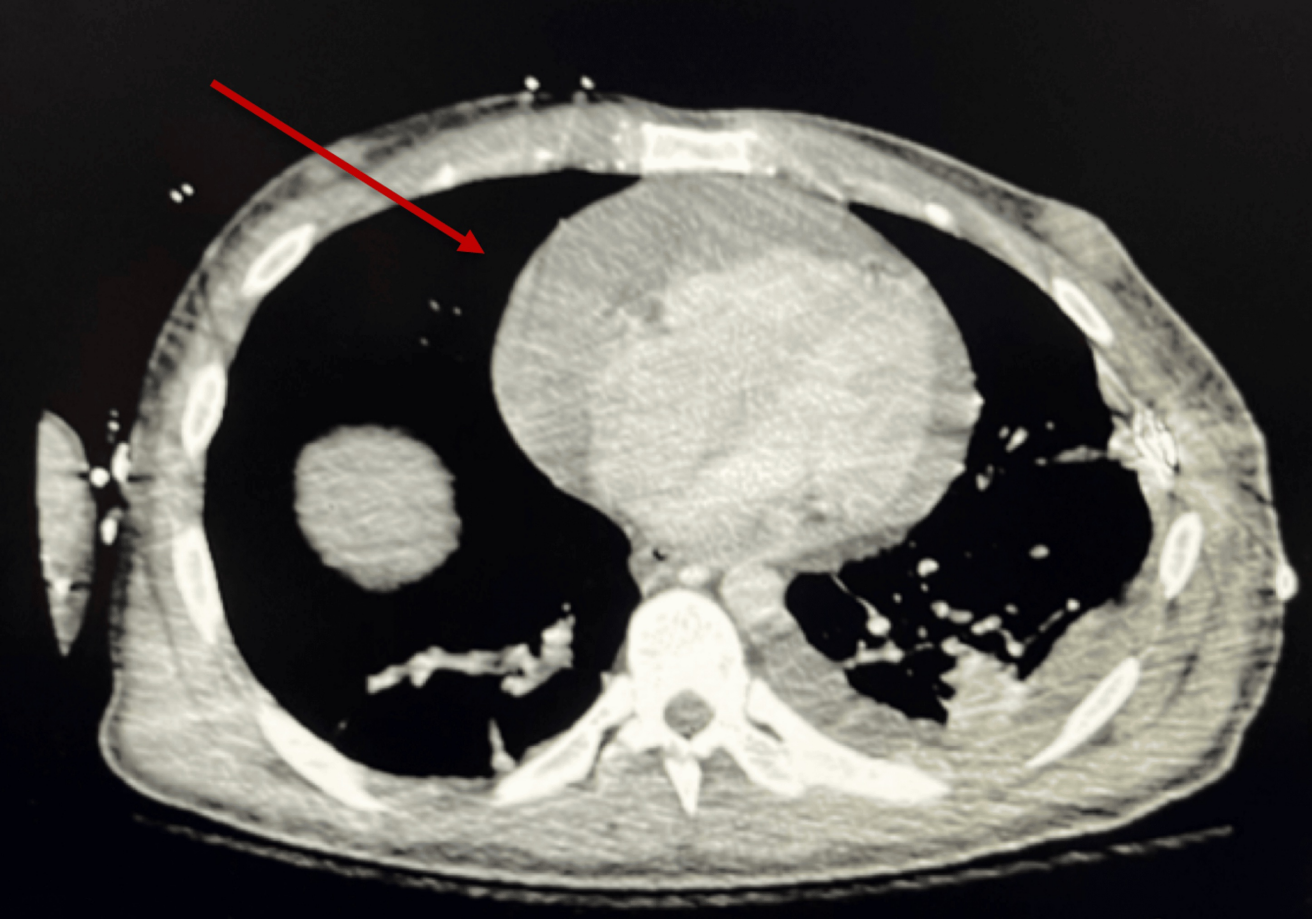
Chest CT 15 days after the initial trauma showing massive pericardial effusion The red arrow shows the accumulation of fluid within the pericardial sac.

Two weeks post-trauma, the patient was hemodynamically stable and had begun working with physical therapy and occupational therapy. However, he suddenly experienced acute hypotension (BP 89/62), severe tachycardia (HR 275), and signs of respiratory distress, raising concerns for an acute pathology. A CT angiography (CTA) was ordered to rule out acute pulmonary embolism. The initial CT of the chest on admission did not show any pericardial effusion (Figure 3); however, the follow-up scan revealed a large pericardial effusion (Figure 4). Stat echocardiography confirmed early tamponade physiology, prompting an urgent consultation with cardiothoracic surgery. The patient was urgently taken to the operating room, where a pericardial window was performed, successfully relieving the cardiac compression.

**Figure 5:**
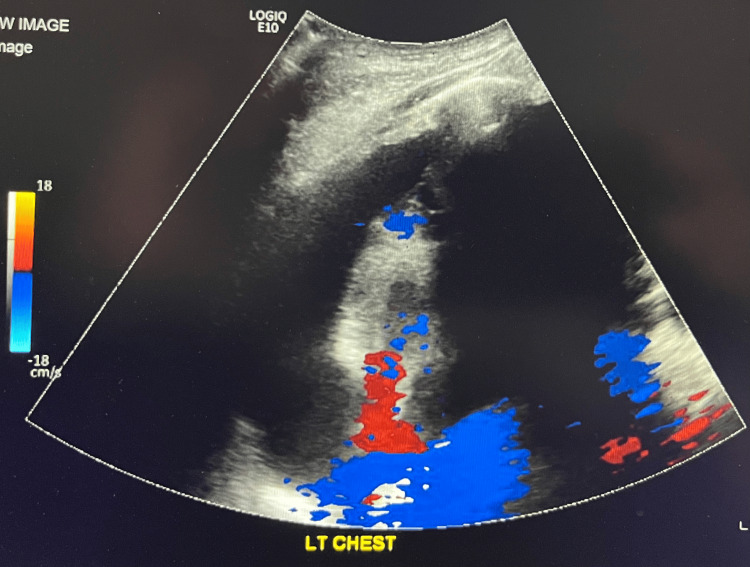
Preoperative ultrasound of the pleural space with color Doppler

**Figure 6:**
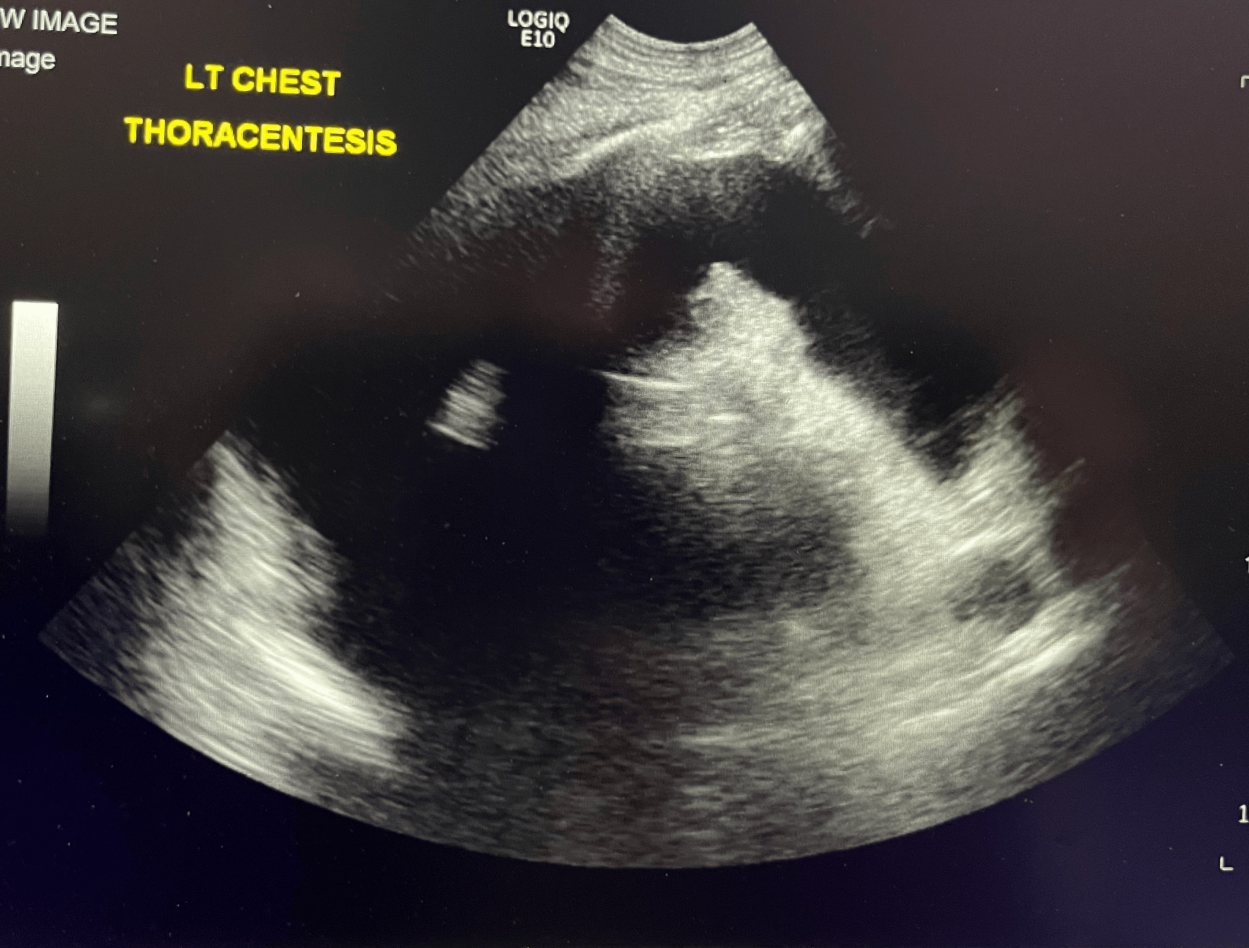
Postoperative ultrasound of the pleural space following interventional radiology drainage

Postoperatively, the patient underwent IR-guided pleural drain placement (Figure 5), resulting in the evacuation of 800 mL of hemorrhagic fluid (Figure 6). This led to hemodynamic stabilization. Since then, the patient has made a significant recovery, as observed during clinic visits with trauma, orthopedics, and cardiothoracic surgery.

## Discussion

Delayed pericardial tamponade following blunt trauma is a rare but potentially life-threatening condition, posing significant diagnostic and management challenges due to its nonspecific presentation and broad differential diagnosis, including myocardial contusion, pulmonary embolism, and hemorrhagic shock. While acute tamponade is typically associated with systolic blood pressure <90 mmHg, subacute presentations often show only mild blood pressure reduction [4].

In blunt trauma, pericardial tamponade often arises from microvascular bleeding, myocardial contusion, or injury to the pericardial vessels. The gradual accumulation of blood or inflammatory exudate within the pericardial sac can progress to clinically significant tamponade, impairing cardiac filling and output. In this case, the progressive accumulation of hemorrhagic fluid was likely exacerbated by the severity of thoracic trauma, as evident from multiple grossly displaced rib fractures and flail segments [5].

Delayed pericardial tamponade is rare, with reported cases presenting days to weeks after trauma. Hermens et al. [5] reported a delayed onset four weeks post-injury; Matthia et al. [4] described a case at nine weeks; and Davey et al. [6] documented tamponade six weeks after rib fractures. These cases highlight the need for continued monitoring, particularly in patients with negative initial imaging.

The delayed presentation complicates the diagnosis, particularly in polytrauma patients with multiple potential causes of hemodynamic instability. In our case, the patient’s change in vitals, e.g., hypotension and tachycardia, prompted evaluation with CTA and echocardiography, confirming a significant pericardial effusion with tamponade physiology. Bedside echocardiography remains a critical diagnostic tool for rapid identification of pericardial fluid and its hemodynamic effects [7].

Management requires prompt pericardial drainage to relieve cardiac compression. Surgical intervention is often necessary in trauma cases to evacuate clotted blood and prevent reaccumulation. In this case, an open pericardial window was performed with evacuation of the clot, leading to hemodynamic improvement. Surgical drainage is more effective in trauma patients than pericardiocentesis alone, as clotted blood often limits its efficacy [3].

This case highlights the need for continued monitoring of trauma patients. While the incidence of delayed tamponade is low, it is potentially fatal and requires early recognition and intervention [8]. Trauma patients with significant thoracic injuries or persistent hemodynamic instability should undergo regular follow-up imaging to detect late-developing pericardial effusion.

## Conclusions

Delayed pericardial tamponade is a rare but potentially fatal complication of blunt thoracic trauma that underscores the importance of continued vigilance even after initial stabilization. This case highlights the complex trajectory that trauma patients may follow, illustrating how life-threatening complications can emerge days to weeks after the primary injury. While the patient initially appeared to be recovering well, the sudden onset of hemodynamic instability due to delayed tamponade exemplifies the unpredictable nature of post-traumatic sequelae. Early recognition remains the cornerstone of effective management. Clinicians must maintain a high index of suspicion, particularly in patients with known blunt chest trauma, elevated cardiac biomarkers, and signs of myocardial injury. Advanced imaging modalities such as CTA and prompt echocardiography are essential tools for diagnosis, while surgical interventions like pericardial windows remain lifesaving.

This case emphasizes the importance of interdisciplinary care, with coordination between trauma surgery, cardiology, cardiothoracic surgery, and critical care teams playing a critical role in successful outcomes. Incorporating ongoing monitoring, follow-up imaging, and patient education into trauma recovery protocols may further aid in the early detection of delayed complications. Ultimately, heightened clinical awareness, timely diagnosis, and proactive management are essential to reducing morbidity and mortality in trauma patients at risk for delayed cardiac complications.
